# Aerobic glycolysis in bladder cancer: research advances and targeted therapy potential

**DOI:** 10.3389/fonc.2026.1799955

**Published:** 2026-04-22

**Authors:** Mengyuan Pan, Tianyi Tao, Dehui Kong, Hua Gong

**Affiliations:** 1 Graduate School, Henan Medical University, Xinxiang, Henan, China; 2 Department of Urology, Shanghai University of Medicine & Health Sciences Affiliated Zhoupu Hospital, Shanghai, China; 3 Graduate School, Shanghai University of Traditional Chinese Medicine, Shanghai, China; 4 Experimental Cellular Therapy Group, University of California, San Francisco, San Francisco, CA, United States

**Keywords:** aerobic glycolysis, bladder cancer, key enzymes, non-coding RNA, signaling pathways, targeted therapy, tumor microenvironment

## Abstract

Bladder cancer (BCa) is one of the most common malignant tumors of the urinary system. Its frequent recurrence, high metastatic potential, and resistance to therapies pose major obstacles to achieving long-term patient survival. As a core feature of tumor metabolic reprogramming, aerobic glycolysis (the Warburg effect) plays an essential role in the development of BCa. Current studies indicate that key glycolytic enzymes such as hexokinase 2 (HK2), pyruvate kinase M2 (PKM2), and lactate dehydrogenase A (LDHA) are abnormally expressed in BCa. These alterations in enzyme activity not only directly reshape energy metabolism but also exert non-metabolic functions, regulating tumor cell proliferation and invasion. Simultaneously, the aberrant activation of signaling pathways such as PI3K/AKT/mTOR and HIF-1α further drives the glycolytic process. Moreover, the lactate produced through glycolysis leads to tumor microenvironment (TME) acidification, which facilitates extracellular matrix remodeling and immune evasion. In terms of treatment, strategies that directly target key glycolytic enzymes and indirectly intervene in the regulation of signaling pathways show promising application potential. Nevertheless, issues related to treatment-associated toxicity and the emergence of therapeutic resistance remain unresolved. This review systematically summarizes the characteristics of key enzymes in aerobic glycolysis, molecular regulatory mechanisms, and advancements in targeted therapy for BCa, aiming to provide new theoretical insights and directions for metabolic intervention and targeted therapy in BCa.

## 1 Introduction

Bladder cancer (BCa) is a common neoplastic disease of the urinary system and remains a leading cause of cancer-related morbidity on a global scale. Data from the Global Cancer Statistics 2022 report estimate approximately 614,000 incident cases and 220,000 deaths attributable to BCa worldwide, placing it among the top ten most frequently diagnosed cancers overall and ranking sixth in incidence among malignancies affecting men (1). The incidence and mortality rates of BCa exhibit significant geographical and demographic disparities, with a notably higher prevalence in developed nations, the elderly population, and males. The primary risk factors include tobacco smoking, occupational chemical exposure, and chronic urinary tract infections (2, 3).

Although substantial progress has been attained in therapeutic strategies through the incorporation of radical surgery, cisplatin-based combination chemotherapy, and immune checkpoint inhibitors, long-term prognosis remains unsatisfactory for many patients (4). The clinical efficacy of these interventions is frequently compromised by the tumor’s high propensity for recurrence and metastasis, as well as the development of intrinsic or acquired resistance to chemotherapeutic agents (5). Furthermore, despite the promise of immunotherapy, a significant subset of patients fails to respond due to immune evasion mechanisms within the tumor microenvironment (6). Consequently, it is essential to further elucidate the mechanisms underlying BCa progression and to identify innovative therapeutic approaches.

Metabolic reprogramming is one of the key features of tumorigenesis, with the Warburg effect being the most representative metabolic abnormality. In 1923, Otto Warburg reported that cancer cells tend to rely on glycolytic metabolism for energy production despite the presence of adequate oxygen, instead of primarily utilizing mitochondrial oxidative phosphorylation (7, 8). Despite the fact that each molecule of glucose in glycolysis generates only 2 molecules of ATP, far fewer than the 30 – 32 molecules generated by oxidative phosphorylation, its ATP production rate is 10 – 100 times faster (9). This high rate and low efficiency metabolic pattern provides a significant survival advantage for cancer cells. On the one hand, the rapidly produced ATP meets the high energy demands of cancer cells; on the other hand, the intermediates of glycolysis provide raw materials for the synthesis of proteins, lipids, and nucleic acids, while the lactate secretion-induced acidification of the microenvironment further promotes extracellular matrix remodeling and immune evasion (10).

Dysregulated expression of key glycolytic enzymes is a defining feature of BCa initiation and progression. Beyond acting as core drivers of energy supply, these enzymes exhibit extensive non-metabolic functions including direct participation in signal transduction, regulation of gene expression, and modulation of immune responses, thereby driving malignant processes such as proliferation, invasion, and drug resistance in a multidimensional manner. Consequently, intervention strategies targeting these key enzymes and their upstream regulatory signaling pathways hold significant promise for clinical translation. This review summarizes the advances on aerobic glycolysis in BCa, focusing on the function, molecular regulatory mechanisms of key glycolytic enzymes, and their interaction with the tumor microenvironment, and also discusses current strategies targeting glycolysis. This paper aims to integrate existing theoretical insights and directions for metabolic intervention and targeted therapy in BCa, and offer research directions and application prospects for future study.

## 2 Glycolytic metabolic characteristics of BCa

Aerobic glycolysis is a hallmark of metabolic reprogramming in BCa and has emerged as an important driver of tumor progression, therapeutic resistance, and microenvironmental remodeling. In BCa, glycolytic activation is not solely determined by changes in individual enzymes, but is shaped by complex regulatory networks and further influenced by disease heterogeneity. This section therefore summarizes the expression and functional roles of major glycolytic enzymes in BCa, outlines the principal mechanisms underlying glycolytic regulation, discusses the emerging contribution of glycogen metabolism to metabolic adaptation, and highlights metabolic differences across BCa subtypes.

### 2.1 Expression and function of key enzymes

Enhanced glycolysis in BCa is driven by abnormal expression and regulation of multiple enzymes and transporters. In addition to supporting metabolic adaptation, these molecules also contribute to tumor progression and treatment response. This section summarizes the major glycolytic enzymes and related transporters implicated in BCa.

#### 2.1.1 Hexokinase

As the initial rate-controlling enzyme in the glycolytic pathway, hexokinase (HK) converts glucose into glucose-6-phosphate (G-6-P), thereby playing a central role in maintaining cellular energy homeostasis. HK comprises four isoforms (HK1–4), of which HK2 shows elevated expression in BCa tissues and is strongly linked to disease progression and clinical outcomes (11). HK2 also exhibits the highest efficiency in promoting aerobic glycolysis. It interacts with voltage-dependent anion channel 1 (VDAC1) on the outer mitochondrial membrane, facilitating the activation of ATP-synthesizing enzymes, thereby enhancing ATP production and inhibiting apoptosis. Besides its metabolic role in glycolysis, HK2 functions as a protein kinase by phosphorylating IκBα; specifically, phosphorylation at the T291 residue of IκBα upregulates programmed death-ligand 1 (PD-L1) expression, enabling cancer cells to evade immune surveillance (12). Moreover, SRC kinase can directly phosphorylate and activate HK2, enhancing both glycolysis and the pentose phosphate pathway (PPP), which in turn promotes nucleotide biosynthesis and increases NADPH production to neutralize cisplatin-induced reactive oxygen species (ROS), ultimately contributing to cisplatin resistance in BCa (13).

#### 2.1.2 Fructose-1-phosphokinase

As the second rate-limiting enzyme of the glycolytic pathway, fructose-1-phosphokinase (PFK-1) catalyzes the ATP-dependent phosphorylation of fructose-6-phosphate (F-6-P) to produce fructose-1,6-bisphosphate (F-1,6-BP). The activity of PFK-1 directly determines the rate of glycolysis. PFK-1 is overexpressed in BCa tissues, and its knockdown significantly suppresses the proliferation, migration, and invasion of BCa cells, while also reducing lactate production and mitigating acidification of the tumor microenvironment (14). The most potent allosteric activator of PFK-1 is fructose-2,6-bisphosphate (F-2,6-BP), which is synthesized by the enzyme 6-phosphofructo-2-kinase/fructose-2,6-bisphosphatase (PFKFB) (15). There are four PFKFB isoforms (PFKFB1 – 4), among which PFKFB3 and PFKFB4 have been shown to promote tumor cell proliferation, invasion, and metastasis by enhancing glycolysis and the PPP. They also regulate autophagy by modulating ROS levels and NADPH production, thereby supporting cancer cell survival and chemoresistance (16). In a recent study on BCa, Qiu et al. demonstrated using single-cell RNA sequencing and immunohistochemistry that PFKFB3 is preferentially upregulated in malignant epithelial cells, particularly in muscle-invasive bladder cancer (MIBC), and is predominantly localized in the nucleus, suggesting a potential role in linking enhanced glycolytic activity to proliferative advantage in aggressive disease. In addition, elevated PFKFB3 expression was significantly associated with poorer overall survival, supporting its value as a prognostic biomarker and a potential metabolic target in BCa (17). Collectively, these results establish a conceptual basis for pursuing therapeutic approaches that target PFKFB in BCa.

#### 2.1.3 Pyruvate kinases

The final rate-limiting step in glycolysis is catalyzed by pyruvate kinase (PK), which facilitates the conversion of phosphoenolpyruvate (PEP) and ADP into pyruvate and ATP. In mammals, there are four isoforms of PK (PKM1, PKM2, PKR, PKL), with PKM2 being markedly overexpressed in a broad spectrum of malignancies and playing a central role in cancer development through metabolic reprogramming, transcriptional regulation, and modulation of the tumor microenvironment (18). Overexpression of PKM2 enhances glucose uptake, lactate production, ATP generation, and cell proliferation in BCa, driving disease progression (19). Signal transducer and activator of transcription 3 (STAT3) plays a key role in BCa cell signaling, and PKM2 interacts with STAT3, promoting its nuclear translocation and activating the HIF-1α/vascular endothelial growth factor (VEGF) pathway to promote angiogenesis in BCa (20). Furthermore, PKM2 dimers can activate STAT3 under the influence of transforming growth factor-β (TGF-β), enhancing PD-L1 transcription and expression, thus promoting immune evasion by BCa cells (21). Recent studies have shown that PKM2 can directly bind to Ubiquitin Carboxyl-terminal Hydrolase L1 (UCHL1), which stabilizes PKM2 by deubiquitinating it, thereby increasing PKM2 levels and promoting BCa cell proliferation, migration, and invasion (22). Moreover, Dihydropyrimidinase-like 2 (DPYSL2) has been found to interact with PKM2, facilitating its transition from an active tetrameric form to an inactive dimeric state, thereby promoting aerobic glycolysis, epithelial-mesenchymal transition (EMT), and BCa progression (23). Environmental carcinogen arsenic promotes PKM2 expression via activation of the GLUT1-mTORC1/HIF-1α signaling axis, while heat shock protein 90 (HSP90) binds to PKM2, increasing its protein stability, thereby synergistically promoting glycolytic reprogramming and tumor progression in BCa (24). These studies highlight the crucial role of PKM2 in BCa, and targeting this key enzyme could hold potential for future therapeutic strategies.

#### 2.1.4 Enolase

Enolase (ENO) is a metal ion-dependent enzyme that plays a key role in the glycolytic pathway by catalyzing the conversion of 2-phosphoglycerate (2-PG) to phosphoenolpyruvate (PEP). In mammals, ENO exists in three isoforms, encoded by the ENO1, ENO2, and ENO3 genes, with ENO1 being overexpressed in various cancers, including BCa, and promoting tumor growth by regulating energy metabolism (25, 26). ENO2 is primarily expressed in neurons and neuroendocrine cells, while ENO3 is mainly expressed in muscle tissue. In BCa tissues, the mRNA and protein levels of ENO1 are significantly higher than those in normal tissues, and its expression correlates positively with tumor grade and clinical stage. Knockdown of ENO1 significantly inhibits BCa cell proliferation and invasion (27, 28). Recent studies revealed that Cell Division Cycle Associated-3 (CDCA3) stabilizes the MYC protein by recruiting tripartite motif containing 28 (TRIM28), and this positive feedback loop upregulates ENO1 expression, promoting BCa glycolysis and progression (29). The transcription factor ARNTL2 directly upregulates ENO1 expression and enhances its enzyme activity through SLC31A1, promoting BCa glycolysis and tumor progression (30). In BCa tissues, N6-methyladenosine (m^6^A) modification levels are significantly elevated, and ENO1 has been identified as a key target gene for m^6^A modification. The RBM15/METTL3 complex increases m^6^A modification on ENO1 mRNA at the 359A site, promoting its translation (31). Furthermore, ENO2 is also highly expressed in BCa. Krüppel-like factor 12 (KLF12) reduces ENO2 expression by binding to the ENO2 promoter to inhibit its transcription (32). The role of ENO3 in BCa remains unclear, but ENO1 and ENO2 are promising targets for future BCa therapies.

#### 2.1.5 Lactate dehydrogenase

Lactate dehydrogenase (LDH) is responsible for catalyzing the interconversion of pyruvate and lactate, marking the final step of the glycolytic pathway. Fattahi et al. reported that serum LDH concentrations are significantly higher in BCa patients than in healthy individuals. Furthermore, these elevated levels correlate with tumor stage and patients’ smoking history, suggesting that LDH may act as a valuable biomarker for both the diagnosis and prognosis of BCa (33). CDKN3 expression is significantly elevated in BCa tissues and promotes tumor cell proliferation, migration, and cisplatin resistance by enhancing glycolytic activity and upregulating LDHA expression. Targeting the CDKN3–LDHA pathway has been suggested as a novel therapeutic strategy to combat cisplatin resistance in BCa (34). Increased LDH activity leads to the excessive production of lactate, which is exported from the cell through monocarboxylate transporter 4 (MCT4), resulting in TME acidification to a pH of 6.0 – 6.5. Lactate, through its acidifying effect on the TME, creates a highly immune-suppressive environment, which helps cancer cells evade immune surveillance (35). Lactate in the extracellular space can also be taken up into cells via monocarboxylate transporter 1 (MCT1). Knockdown of MCT1 reduces the expression of key glycolytic enzymes and lactate secretion, thereby inhibiting BCa cell proliferation, migration, and invasion. Consequently, targeting MCT1 may represent a novel approach to improving BCa therapy (36).

#### 2.1.6 Glucose transporter

Glucose transporter (GLUT) proteins are widely expressed in various human tissues and are responsible for mediating the transmembrane transport of glucose, serving as the fundamental prerequisite for cellular glycolysis. They are highly expressed in multiple cancers and are positively correlated with the degree of malignancy (37). Currently, several isoforms of GLUT (GLUT1-14) have been identified, with GLUT1 being upregulated in BCa tissues, where its levels rise alongside tumor progression. Abnormal glycosylation of GLUT1, particularly sialyl-Tn (STn) modification, enhances its membrane localization and glucose uptake capacity, promoting tumor growth and correlating with the invasiveness of BCa (38). Phosphatidylinositol glycan anchor biosynthesis class T (PIGT) is upregulated in BCa, and it drives GLUT1 glycosylation to promote BCa proliferation, metastasis, and metabolic reprogramming. Targeting the PIGT-GLUT1 pathway may provide a potential therapeutic strategy for BCa (39). GLUT3 is also upregulated in BCa, and studies have shown that YTH domain containing 1 (YTHDC1) inhibits GLUT3 expression, suppressing glycolysis and BCa progression. Moreover, GLUT3 upregulates RING finger protein 183 (RNF183), leading to the degradation of YTHDC1, forming a positive feedback loop that exacerbates BCa progression (40). Recent evidence indicates that GLUT4 is highly expressed in BCa and is positively correlated with fibroblast activation protein (FAP) expression in cancer-associated fibroblasts (CAFs) and increased intratumoral microvessel density, suggesting that GLUT4-associated metabolic reprogramming may be linked to stromal activation and angiogenesis during BCa progression (41). GLUT1, GLUT3, and GLUT4 all play key roles in BCa progression, and targeting GLUT transporters could be a potential focus for future BCa research.

#### 2.1.7 Other key enzymes in the glycolytic pathway

Other key enzymes in the glycolytic pathway also play critical roles in BCa progression. Glycolytic phosphoglycerate kinase 1 (PGK1) is overexpressed in BCa tissues and positively correlates with Ki-67 expression. Functional studies further showed that PGK1 promotes glucose uptake and lactate production, thereby enhancing BCa cell proliferation, migration, EMT-related progression, and cisplatin resistance, supporting its potential as a prognostic biomarker and therapeutic target in BCa (42). Aldolase A (ALDOA) has been shown to activate the EGFR-MAPK/AKT pathway by inhibiting E-cadherin, thereby driving EMT and BCa progression. Recent studies indicate that ALDOA may be a key regulator of BCa invasion and metastasis (43). Zhu et al. quantified the relationship between glyceraldehyde-3-phosphate dehydrogenase (GAPDH) activity and glycolytic flux in cancer cells with the Warburg phenotype, showing that glycolysis remains relatively stable until GAPDH activity falls below a critical threshold, beyond which disruption of the coupled GAPDH–PGK1 reaction leads to a marked decline in glycolytic flux, thereby providing a mechanistic basis for GAPDH-targeted metabolic intervention (44). Phosphoglycerate mutase 1 (PGAM1) has been implicated in BCa progression, and knockdown of PGAM1 results in the accumulation of 3-phosphoglycerate (3-PG) and a reduction in 2-phosphoglycerate (2-PG), which inhibits glycolysis and the PPP, ultimately suppressing cell proliferation and inducing apoptosis. Targeting PGAM1 may represent a novel therapeutic strategy for BCa (45). Additionally, Triosephosphate isomerase 1 (TPI1) has been found to be overexpressed in BCa, and beyond its metabolic role, it functions as a scaffold protein, forming a complex with AKT and MDM2, enhancing AKT phosphorylation at Ser166 and promoting the ubiquitination and degradation of p53, thus driving BCa progression (46).

**Figure 1:**
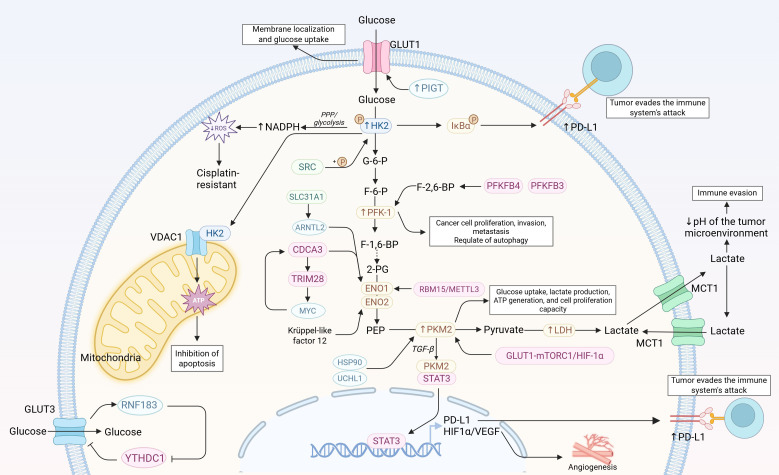
Key enzymes of aerobic glycolysis and their regulatory roles in bladder cancer progression. Created with BioRender.com.

Collectively, these findings indicate that glycolytic enzymes in BCa function not only as metabolic catalysts but also as important regulators of tumor progression and treatment response. Beyond sustaining energy production, the dysregulation of these enzymes converges on several recurring mechanistic themes. First, enhanced glycolytic flux is closely linked to chemoresistance, partly by supplying metabolic intermediates that support redox homeostasis, antioxidant defense, and adaptive survival under therapeutic stress (47). Second, increased lactate production contributes to immune modulation by promoting an acidic and immunosuppressive tumor microenvironment, thereby impairing antitumor immune activity and facilitating tumor cell survival (48). In addition, lactate-related epigenetic regulation, particularly protein lactylation, has emerged as another mechanism through which glycolytic reprogramming may reshape transcriptional programs and further drive BCa progression (49, 50). Taken together, these observations suggest that glycolysis is closely integrated with multiple malignant features of BCa. **Figure 1** summarizes the major glycolytic enzymes and related regulators in BCa and highlights their associations with tumor progression, immune evasion, angiogenesis, and chemoresistance.

### 2.2 Molecular mechanisms of glycolysis regulation

#### 2.2.1 Signaling pathways

The PI3K/AKT/mTOR pathway is one of the key metabolic pathways that induce and regulate the Warburg effect, and critically contributes to tumorigenesis, progression, and therapeutic response (51). Activation of PI3K/AKT drives tumor growth by inhibiting apoptosis and promoting cell cycle progression, while NOTCH3 promotes transcription by binding to the C-promoter binding factor 1 (CSL) element in the secreted phosphoprotein 1 (SPP1) promoter, enhancing PI3K and AKT phosphorylation levels and thereby advancing BCa progression (52). EMT and angiogenesis are two major factors contributing to BCa metastasis. Studies have shown that TEA domain transcription factor 4 (TEAD4) and RNA polymerase III subunit G (POLR3G) can induce EMT by activating the PI3K/AKT pathway (53, 54). Fibroblast growth factor 6 (FGF6) is highly expressed in BCa, and it promotes the expression of glycolytic enzymes by activating the PI3K/AKT pathway. This leads to enhanced glycolysis and lactate accumulation, which further stimulates the secretion of angiogenesis factors such as VEGF and erythropoietin (EPO), driving BCa metastasis (55). The key proline metabolic enzyme pyrroline-5-carboxylate reductase 1 (PYCR1) is overexpressed in BCa, and it directly interacts with EGFR, thereby activating the PI3K/AKT signaling pathway. This activation leads to the upregulation of glycolytic enzymes like GLUT1, HK1, and LDHA, as well as increased lactate production, thereby promoting BCa progression (56). The tumor suppressor phosphatase and tensin homolog (PTEN) regulates the PI3K/AKT pathway, and its loss results in continuous activation of this pathway, promoting cisplatin resistance. YTHDC1 stabilizes PTEN mRNA through an m^6^A-dependent mechanism, maintaining PTEN expression and enhancing BCa sensitivity to cisplatin, providing a novel therapeutic target to overcome resistance (57). The downregulation of major histocompatibility complex class I (MHC-I) leads to immune escape by preventing cytotoxic T lymphocytes (CTLs) from recognizing and eliminating tumor cells. Ras GTPase-activating protein SH3 domain-binding protein 1-splicing factor 7 (G3BP1-SLU7) complex stabilizes the class IA PI3Ks mRNA, promoting its translation and activation of the PI3K/AKT pathway, downregulating MHC-I, and promoting immune evasion. This discovery offers novel theoretical support for precision immunotherapy in BCa (58). Moreover, hepatitis B X-interacting protein (HBXIP) has been confirmed as a key molecule connecting metabolism with angiogenesis in BCa. It activates the AKT/mTOR pathway, promoting the expression of glycolytic enzymes and lactate production, which in turn upregulates VEGF and EPO, facilitating tumor angiogenesis (59). mTOR is a central downstream effector of the PI3K/AKT pathway, and its two distinct complexes, mTORC1 and mTORC2, respectively regulate cellular metabolism and proliferation, as well as invasion and metastasis. Although mTOR is a key regulator in BCa, targeting it has not significantly improved patient prognosis. Future efforts could focus on optimizing mTOR-targeted therapies through combination treatments and overcoming drug resistance (60).

The Wnt/β-catenin and MAPK signaling pathways also make critical contributions to the metabolic reprogramming of BCa. Studies have confirmed that scaffold matrix attachment region binding protein 1 (SMAR1) inhibits Wnt/β-catenin pathway activity, thereby suppressing BCa cell proliferation, EMT, and the Warburg effect (61). FGF6 activates the MAPK signaling pathway and enhances key glycolytic enzyme expression, promoting aerobic glycolysis and angiogenesis in BCa (55).

#### 2.2.2 Hypoxia-inducible factor-1α

Hypoxia-inducible factor-1α (HIF-1α) is one of the most critical transcription factors, and its function is closely related to the metabolic reprogramming of BCa. HIF-1α regulates the transcription of numerous genes, including GLUT1, HK2, PFK1, PKM2, LDHA, and EPO, thereby promoting glycolysis and lactate production. Inhibition of HIF-1α has been shown to be an effective strategy for treating BCa, specifically by suppressing glycolysis and improving cisplatin resistance. Zhang et al. discovered that calponin 1 (CNN1) downregulates the expression of key glycolytic enzymes GLUT1, PKM2, and LDHA by inhibiting the HIF-1α-PDK1 axis, thus blocking glycolysis and suppressing BCa progression (62). HIF-1α plays a significant role in BCa cisplatin resistance through multiple mechanisms. Yu et al. found that HIF-1α induces the expression of miR-424 in response to cisplatin, which, in turn, suppresses the pro-apoptotic genes unc-5 netrin receptor B (UNC5B) and sirtuin 4 (SIRT4), helping BCa cells evade cisplatin-induced apoptosis and thereby acquire resistance (63). Shigeta et al. showed that isocitrate dehydrogenase 2 (IDH2) stabilizes HIF-1α by catalyzing the production of 2-hydroxyglutarate (2-HG), which inhibits prolyl hydroxylase domain containing 2 (PHD2) activity. The reduced activity of PHD2 decreases HIF-1α degradation, stabilizing HIF-1α, activating the PPP, and promoting the generation of NADPH and glutathione (GSH), thus enhancing the antioxidant defense and ultimately leading to cisplatin resistance (64). Additionally, hypoxia reduces EGLN2 inhibition of nuclear factor kappa B (NF-κB), activating the positive feedback loop between MUC1 C-terminal subunit (MUC1-C) and HIF-1α, which jointly drive glycolysis and contribute to gemcitabine resistance (65). These studies identify potential therapeutic targets for overcoming cisplatin resistance in BCa, highlighting the need for further investigation.

#### 2.2.3 Non-coding RNA

Non-coding RNA (ncRNA) is a category of RNA transcripts that do not encode proteins but possess significant regulatory functions. Major subtypes, including microRNA (miRNA), long non-coding RNA (lncRNA), and circular RNA (circRNA), influence tumor cell energy metabolism by regulating key glycolytic enzymes or signaling pathways, thereby promoting the progression of BCa (66). Among the miRNA family, miR-125b-5p directly suppresses the expression of HK2 by binding to the 3’-UTR of HK2 mRNA, resulting in decreased HK2 protein expression. The downregulation of HK2 further weakens the phosphorylation activation of the PI3K/AKT pathway, ultimately suppressing glycolysis in BCa cells and promoting apoptosis (67). lncRNA and circRNA act as “miRNA sponges,” exerting their functions by sequestering miRNA (68). Xiang et al. reported that lncRNA small nucleolar RNA host gene 1 (SNHG1) promotes BCa cell proliferation by sequestering miR-143-3p, relieving its inhibition on HK2, thus enhancing glycolysis (69). circRNA has also been shown to participate in BCa progression by regulating key glycolytic enzymes. Zhong et al. discovered that hsa_circ_0000235 (circ235) relieves the suppression of miR-330-5p on MCT4, upregulating its expression, enhancing glucose uptake and lactate production, and promoting BCa progression (70). Zuo et al. found that circRNA ST6 N-acetylgalactosaminide α-2,6-sialyltransferase 6 (circST6GALNAC6) is downregulated in BCa, and its overexpression inhibits glycolysis and BCa growth by promoting the degradation of HK1 through the recruitment of RNA-binding protein fused in sarcoma (FUS), which enhances the mRNA stability of Parkin RBR E3 ubiquitin protein ligase (PRKN) (71). CircXRN2 stabilizes large tumor suppressor kinase 1 (LATS1) to activate the Hippo pathway, thereby inhibiting glycolysis, lactate production, and downstream H3K18 lactylation, ultimately downregulating the expression of the oncogene lipocalin 2 (LCN2) and inhibiting BCa progression (72). Further studies have also revealed the value of lncRNA in BCa subtyping and prognosis. Mao et al. identified four glycolysis-associated lncRNA-regulated subtypes of BCa, among which the C1 subtype exhibited the highest glycolysis score and the poorest prognosis, and further developed a three-lncRNA signature for prognostic stratification (73). As research into ncRNA continues to deepen, it provides new theoretical insights and potential targets for precision therapy in BCa.

#### 2.2.4 Epigenetic modifications

**Figure 2:**
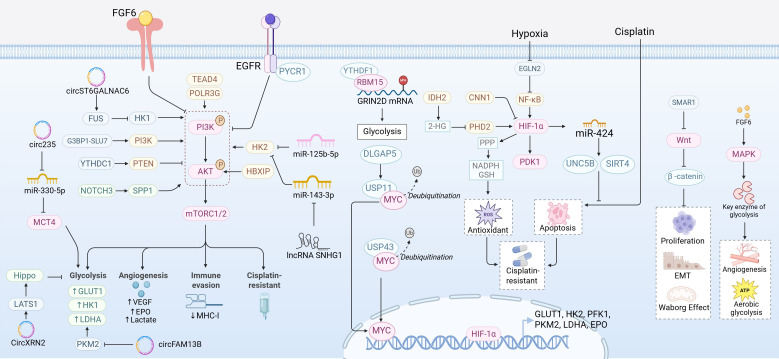
Molecular regulatory networks of aerobic glycolysis in bladder cancer: signaling pathways, transcription factors, non-coding RNAs, and epigenetic modifications. Created with BioRender.com.

Epigenetic modifications regulate the stability of key proteins and histone modifications, finely controlling the metabolic reprogramming in BCa. At the protein stability level, Li et al. found that the deubiquitinase ubiquitin-specific protease 43 (USP43) directly binds to and deubiquitinates MYC, enhancing its stability and promoting the expression of key glycolytic genes, thus facilitating glycolysis and promoting BCa metastasis (74). Deng et al. reported that discs large associated protein 5 (DLGAP5) stabilizes MYC expression through USP11-mediated deubiquitination, which in turn enhances glycolysis and promotes gemcitabine resistance (75). At the RNA transcriptional level, m^6^A readers like YTH N6-methyladenosine RNA binding protein 1 (YTHDF1), in combination with RNA binding motif protein 15 (RBM15), form a regulatory axis that enhances the stability and translation efficiency of glutamate ionotropic receptor NMDA type subunit 2D (GRIN2D) mRNA, directly driving glycolysis in BCa cells (76). Recent studies have further confirmed the prognostic value of m^6^A in BCa. Zhou et al. integrated m^6^A modification with glycolytic genes to construct a risk model based on inositol pentakisphosphate 2-kinase 2 (IP6K2) and phospholipase A2 group IIF (PLA2G2F), which not only predicts patient survival but also identifies that high-risk groups have elevated immune checkpoint expression and a poor response to immunotherapy (77). In terms of histone modifications, H3K18 lactylation, driven by glycolytic product lactate, directly activates the expression of transcription factors Yin Yang 1 (YY1) and Y-box binding protein 1 (YBX1), representing a critical epigenetic mechanism that contributes to cisplatin resistance in BCa (78). Collectively, these findings indicate that aerobic glycolysis in BCa is regulated by multiple upstream mechanisms. **Figure 2** summarizes the major regulatory networks involved in this process, including signaling pathways, transcription factors, non-coding RNAs, and epigenetic modifications.

#### 2.2.5 Glycogen metabolism

In addition to canonical glycolytic pathways, glycogen metabolism has also emerged as a potential metabolic dependency in BCa. As the major intracellular storage form of glucose, glycogen serves as a dynamic energy reserve that may support tumor survival and adaptation under nutrient-limited conditions in the tumor microenvironment (TME). Studies have shown that glycogen phosphorylase (PYGL) and other glycogen-degrading enzymes are highly expressed in BCa and are associated with tumor invasiveness. Under metabolic stress, PYGL mobilizes glycogen stores by converting them into glucose-1-phosphate, which can subsequently enter the glycolytic pathway to sustain ATP production (79).

Recent studies have further identified BCa-specific vulnerabilities related to glycogen remodeling. Loss of the glycogen debranching enzyme amylo-α-1,6-glucosidase, 4-α-glucanotransferase (AGL) has been implicated in BCa progression. AGL deficiency disrupts normal glycogen breakdown and promotes metabolic rewiring, shifting BCa cells toward increased glucose dependence and enhanced serine/glycine biosynthesis to support proliferation (80). Notably, the oncogenic relevance of glycogen remodeling is not limited to BCa. In lung adenocarcinoma, glycogen accumulation has been associated with higher tumor grade and poorer prognosis, and spatial metabolomics has shown that glycogen is enriched within tumor regions. Moreover, dietary or genetic interventions that increase glycogen levels can accelerate lung adenocarcinoma progression (81). Together, these findings suggest that glycogen metabolism may represent a complementary metabolic pathway supporting tumor growth, and that targeting key nodes in this process could provide additional therapeutic opportunities, although the underlying mechanisms are likely to vary across cancer types (82).

### 2.3 Glycolytic heterogeneity in bladder cancer across molecular and clinical subtypes

BCa is a heterogeneous disease in which non-muscle-invasive bladder cancer (NMIBC) and MIBC differ markedly in their clinical behavior and metabolic characteristics (83). Overall, progression from NMIBC to MIBC is generally accompanied by increased aerobic glycolysis, consistent with the greater metabolic demands associated with tumor invasion and progression.

In terms of glycolytic enzyme expression and regulatory mechanisms, MIBC tends to exhibit a more pronounced Warburg effect than NMIBC (11, 84). Clinical transcriptomic studies have shown that multiple key glucose transporters and glycolytic enzymes, such as GLUT1, HK2, PFKFB3, and LDHA, are more frequently upregulated in MIBC, and that their elevated expression is associated with advanced tumor stage, higher histological grade, and poorer survival (11, 17, 85). This difference in glycolytic activity is attributable not only to variations in enzyme abundance, but also to heterogeneity in the underlying regulatory programs. At the molecular level, MIBC can be broadly classified into basal/squamous and luminal subtypes, each with distinct metabolic features (86). Basal-like tumors are more commonly associated with hypoxia, HIF-1α activation, and EGFR/c-Myc signaling, and accordingly tend to display a more pronounced glycolytic phenotype (87). In contrast, luminal tumors, shaped by transcriptional regulators such as PPARγ, FOXA1, and GATA3, appear to be less dependent on glycolysis and may retain a greater capacity for oxidative and lipid metabolism (88).

These differences may also have important therapeutic implications. Because basal-like MIBC appears to depend more heavily on glycolysis, it may represent a more suitable context for glycolysis-targeted intervention. This possibility is particularly noteworthy in light of evidence linking enhanced glycolysis to cisplatin resistance, thereby providing a rationale for combining metabolic inhibitors with neoadjuvant chemotherapy in invasive BCa (89). In contrast, the therapeutic context of NMIBC is distinct, as its management primarily relies on intravesical Bacillus Calmette-Guérin (BCG) immunotherapy following transurethral resection (90). Because an effective BCG response requires glycolysis-supported activation of local immune cells, broad inhibition of glycolytic flux in this setting could potentially weaken antitumor immunity (91). Taken together, these findings suggest that the development of glycolysis-targeted strategies in BCa should take both molecular and clinical subtype heterogeneity into account, rather than adopting a one-size-fits-all approach.

## 3 Glycolysis and tumor microenvironment interactions

The tumor microenvironment (TME) is an intricate ecosystem composed of cancer cells, metabolites such as lactate, the extracellular matrix, and neighboring stromal cells, including immune cells, fibroblasts, and endothelial cells. The formation of this ecosystem is closely associated with glycolysis. Tumor cells secrete large amounts of lactate through the Warburg effect, and lactate, by acidifying the TME, promotes cancer cell migration and angiogenesis, while simultaneously inhibiting immune cell function, aiding in immune evasion by the tumor (92). Pyrroline-5-carboxylate reductase 1 (PYCR1) is a key molecule connecting BCa metabolism with the TME. On one hand, it enhances glycolysis directly via the EGFR/PI3K/AKT pathway (56); on the other hand, in the hypoxic TME, the lactate produced by PYCR1 acts as a substrate for histone lactylation, which upregulates the transcription of SLC6A14, enhancing glutamine metabolism and promoting the malignant progression of BCa (93). Lv et al. found that circular RNA family with sequence similarity 13 member B (circFAM13B) inhibits glycolysis and lactate production by reducing PKM2 stability, thus lowering TME acidity and enhancing the cytotoxic function of CD8^+^ T cells, which improves the efficacy of PD-1 blockade in BCa therapy. This finding offers new potential targets for combination therapy in BCa (94). Additionally, SIRT6 deficiency promotes lactate secretion and TME acidification via activation of the UHRF1/MCT4 axis, impacting immune cell function (95). CAFs can also promote tumor progression and immune evasion by secreting cytokines and remodeling the extracellular matrix. Zhang et al. discovered that CAFs secrete C-X-C motif chemokine ligand 12 (CXCL12) to activate the JAK2/STAT3 pathway, leading to the upregulation of the deubiquitinase cylindromatosis (CYLD). This reduces P62 ubiquitination and causes P62 accumulation, which subsequently inhibits the autophagic degradation of PD-L1. Consequently, increased PD-L1 expression on the cell surface inhibits T cell activity and facilitates immune escape in BCa (96).

Hypoxia is a defining feature of the TME, and HIF-1α is activated under hypoxic conditions. It promotes tumor cell proliferation, invasion, angiogenesis, and lymph node metastasis by targeting and regulating the vascular endothelial growth factor A (VEGFA) gene (97). Furthermore, hypoxia further enhances glycolysis through signaling pathways, including HIF-1α. Luo et al. found that p53 mutations and hypoxia work synergistically to promote glycolysis and the PPP, leading to TME acidification and immune suppression, such as increased Treg cell infiltration. This creates a glycolysis-acidification-immune suppression positive feedback loop, which drives the progression of BCa (98). Targeting and regulating the hypoxic signaling pathways and metabolic reprogramming in the TME may provide novel intervention strategies for BCa treatment. Additionally, the mechanical properties of the TME also influence the metabolic phenotype. Studies have shown that tumor cells in BCa with an inherent soft tumor cell characteristic can sense the soft extracellular matrix, activating the ITGB8/TRIM59/AKT/mTOR signaling axis. This significantly upregulates key glycolytic enzymes such as GLUT1, HK2, and PKM2, linking the mechanical microenvironment directly to aerobic glycolysis (99).

## 4 Targeted therapeutic strategies for glycolysis in BCa

**Figure 3:**
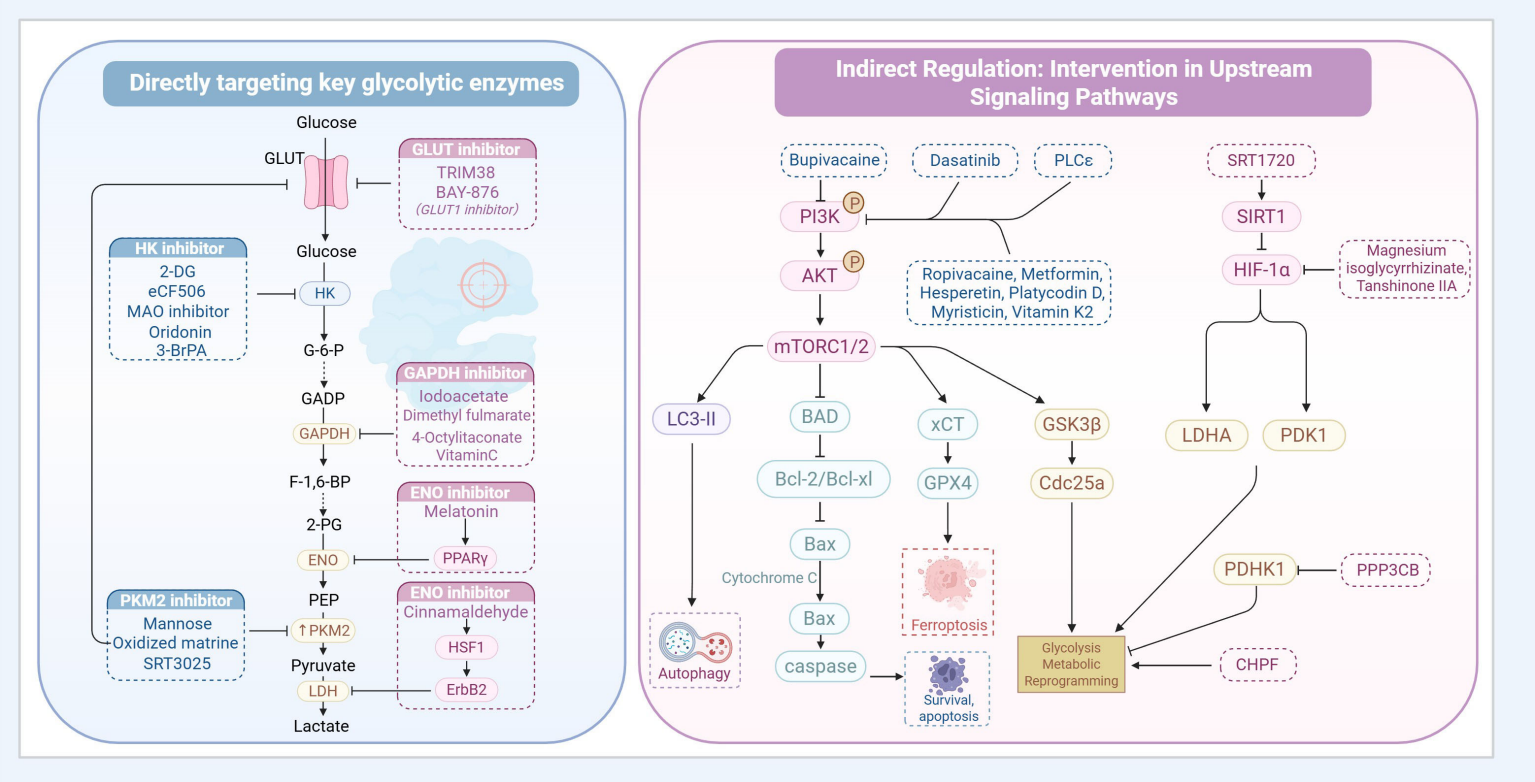
Therapeutic strategies targeting aerobic glycolysis in bladder cancer: direct enzyme inhibition and indirect pathway intervention. Created with BioRender.com.

Based on the expression and functional characteristics of key enzymes in the glycolytic pathway, such as HK, PKM2, and LDH, targeting these molecular nodes to disrupt energy supply, inhibit tumor proliferation, and potentiate chemotherapy sensitivity has emerged as a potential breakthrough for BCa treatment. The currently reported glycolysis-targeting agents and metabolism-related pathway modulators in BCa are summarized in **Table 1**. **Figure 3** outlines the overall therapeutic framework by distinguishing two major strategies: direct targeting of key glycolytic enzymes and indirect modulation of upstream regulatory pathways. The following discussion focuses on these two categories.

**Table 1:** Representative glycolysis-targeting agents and metabolism-related pathway modulators in bladder cancer.

Category	Target/pathway	Therapeutic inhibitor/agent	Brief mechanism of action	Developmental stage in BCa	Reference(s)
Direct glycolytic targeting	HK2	2-Deoxy-D-glucose	A glucose analog that competitively inhibits hexokinase-mediated glucose phosphorylation and suppresses glycolytic flux.	*In vitro*/*in vivo*	(11)
Direct glycolytic targeting	SRC-HK2	eCF506	Reverses cisplatin resistance by downregulating HK2 expression and suppressing glycolysis-associated metabolic reprogramming.	*In vitro*/*in vivo*	(13)
Direct glycolytic targeting	MAO/HK2	Pargyline	Indirectly inhibits HK2-associated glycolytic activity by modulating metabolic flux and glucose-dependent energy production.	*In vitro*	(100)
Direct glycolytic targeting	HK1	Oridonin	Covalently targets HK1 at Cys-813, inhibits glycolysis, and enhances the antitumor efficacy of anti-PD-L1 immunotherapy.	*In vitro*/*in vivo*	(101)
Direct glycolytic targeting	PKM2	Mannose	Directly interacts with PKM2, reduces lactate production and lactylation, and induces pyroptosis through NF-κB pathway activation.	*In vitro*/*in vivo*	(102)
Direct glycolytic targeting	PKM2	Oxymatrine	Inhibits PKM2-mediated glycolysis, downregulates GLUT1, reduces glucose uptake and lactate production, and enhances cisplatin sensitivity.	*In vitro*	(103)
Direct glycolytic targeting	PKM2	SRT3025-loaded hybrid liposomes	Downregulates PKM2 and cooperates with oxaliplatin to suppress metabolic reprogramming and improve chemosensitivity.	*In vitro*/*in vivo*	(104)
Direct glycolytic targeting	GLUT1	BAY-876	Inhibits GLUT1-dependent glucose uptake and suppresses tumor growth, particularly in BCa with low TRIM38 expression.	*In vitro*/*in vivo*	(105)
Direct glycolytic targeting	ENO1	Melatonin	Reduces ENO1 expression via the PPARγ/ENO1 pathway, suppresses glycolysis, and potentiates the efficacy of gemcitabine.	*In vitro*/*in vivo*	(106)
Direct glycolytic targeting	LDHA	Cinnamaldehyde	Inhibits ErbB2/HSF1/LDHA signaling, thereby reducing LDHA expression/activity and suppressing glycolysis.	*In vitro*	(107, 108)
Direct glycolytic targeting	GAPDH/HK2	3-Bromopyruvate	Broadly inhibits glycolysis through GAPDH and HK2 suppression, thereby inducing apoptosis and necrosis in highly malignant BCa cells.	*In vitro*	(109)
Indirect pathway modulation	PI3K/AKT/mTOR	Bupivacaine	Inhibits PI3K/AKT/mTOR signaling, leading to apoptosis, ferroptosis, and suppression of BCa growth.	*In vitro*/*in vivo*	(110)
Indirect pathway modulation	SRC/PI3K/AKT/mTOR	Dasatinib	Suppresses Src-dependent PI3K/AKT/mTOR signaling, thereby inducing apoptosis, autophagy, and growth inhibition in BCa cells.	*In vitro*	(111)
Indirect pathway modulation	ALOX5/PI3K/AKT	Deoxyschizandrin	Targets ALOX5 and suppresses PI3K/AKT phosphorylation, thereby inhibiting proliferation, migration, and invasion while promoting apoptosis.	*In vitro*	(112)
Indirect pathway modulation	PI3K/AKT	Ropivacaine	Inhibits PI3K/AKT signaling and suppresses proliferation and invasion while promoting apoptosis and autophagy.	*In vitro*/*in vivo*	(113)
Indirect pathway modulation	PI3K/AKT/mTOR	Metformin	Inhibits PI3K/AKT/mTOR signaling and suppresses BCa cell migration and growth while promoting apoptosis.	*In vitro*	(114)
Indirect pathway modulation	PI3K/AKT	Hesperetin	Suppresses SRC/PI3K/AKT signaling while promoting apoptosis and ferroptosis, thereby inhibiting BCa cell proliferation and migration.	*In vitro*	(115)
Indirect pathway modulation	PI3K/AKT	Platycodin D	Inactivates ROS-dependent PI3K/AKT/mTOR signaling to induce apoptosis and DNA damage in BCa cells.	*In vitro*	(116)
Indirect pathway modulation	ESR1/PI3K/AKT	Cardamomin	Upregulates ESR1 to inhibit PI3K/AKT signaling, leading to apoptosis, G0/G1 arrest, and suppression of BCa proliferation, invasion, and tumorigenesis.	*In vitro*/*in vivo*	(117)
Indirect pathway modulation	SIRT1/HIF-1α	SRT1720	Activates SIRT1 to deacetylate HIF-1α and repress the hypoxia pathway, thereby suppressing BCa organoid growth and tumor progression.	Organoid/*in vivo*	(118)
Indirect pathway modulation	PI3K/AKT/HIF-1α/AMPK	Vitamin K2	Promotes PI3K/AKT/HIF-1α-mediated glycolysis under glucose-limited conditions, leading to AMPK-dependent autophagic cell death.	*In vitro*	(119)
Indirect pathway modulation	HIF-1α	Tanshinone IIA	Downregulates Aurora A, HIF-1α, and Bcl-2 to promote apoptosis and suppress BCa progression.	*In vitro*/*in vivo*	(120)
Indirect pathway modulation	NF-κB/HIF-1α	Magnesium isoglycyrrhizinate	Upregulates miR-26b to repress Nox4 and downstream NF-κB/HIF-1α signaling, thereby inhibiting BCa progression and promoting apoptosis.	*In vitro*/*in vivo*	(121)

BCa, bladder cancer; HK, hexokinase; PKM2, pyruvate kinase M2; GLUT1, glucose transporter 1; ENO1, enolase 1; LDHA, lactate dehydrogenase A; MAO, monoamine oxidase.

### 4.1 Direct targeting of key glycolytic enzymes

In BCa treatment, several inhibitors targeting HK have shown significant potential. 2-deoxy-D-glucose (2-DG), a classic competitive inhibitor, directly blocks the glucose phosphorylation activity of HK2 (11). The SRC kinase inhibitor eCF506 reverses cisplatin resistance by downregulating HK2 expression (13). Monoamine oxidase (MAO) inhibitors such as pargyline indirectly inhibit HK2 activity by modulating metabolic flux (100). In addition to HK2, recent studies have found that the natural product oridonin acts as a potent covalent inhibitor of HK1. It inhibits the enzyme’s activity by targeting cysteine at position 813 (Cys-813), blocking glycolysis and enhancing the effectiveness of anti-PD-L1 immunotherapy (101). These inhibitors offer diverse strategies for targeting HK and provide potential avenues for BCa glycolysis-targeted therapy.

Mannose interacts directly with PKM2, suppressing its enzymatic function and lowering lactate levels, thereby blocking lactylation modifications, promoting PKM2 nuclear translocation, and ultimately activating the NF-κB pathway to induce tumor cell pyroptosis (102). Additionally, oxidized matrine also inhibits PKM2-mediated glycolysis, downregulating GLUT1 expression, reducing glucose uptake and lactate production, inhibiting BCa cell proliferation, migration, and invasion, and enhancing cisplatin sensitivity (103). Sirtuin 1 activator 3025 (SRT3025) targets PKM2 expression through a nanodelivery system and synergistically downregulates the PI3K/AKT/mTOR pathway along with oxaliplatin, improving chemotherapy sensitivity (104). These three agents exert antitumor effects by intervening in PKM2-mediated metabolic reprogramming.

Research demonstrates that TRIM38 inhibits the progression of BCa through facilitating the ubiquitination and degradation of GLUT1. Therefore, using GLUT1 inhibitors such as BAY-876 in BCa with low TRIM38 expression can effectively inhibit tumor growth (105). Melatonin significantly reduces ENO1 expression through the PPARγ/ENO1 signaling pathway, suppressing glycolysis in BCa cells and enhancing the antitumor effect of chemotherapy drugs like gemcitabine (106). Studies have revealed the potential of cinnamon and its active components in targeting glycolysis. Cinnamaldehyde and its water extract can inhibit ErbB2/HSF1/LDHA signaling, downregulating LDHA expression and activity, thereby inhibiting glycolysis in BCa cells (107, 108). The halogenated pyruvate derivative 3-bromopyruvate (3-BrPA), a glycolysis inhibitor, selectively induces apoptosis and necrosis in highly malignant BCa cells by inhibiting GAPDH and HK2 activity (109).

### 4.2 Indirect regulation: interventions of upstream pathway

Several drugs have been identified that inhibit BCa development by regulating the PI3K/AKT/mTOR signaling pathway and its downstream targets. For example, bupivacaine can inhibit the PI3K/AKT/mTOR pathway, downregulate anti-apoptotic protein B-cell lymphoma 2 (Bcl-2), upregulate pro-apoptotic protein Bax, and increase cytochrome C levels, while simultaneously reducing the expression of solute carrier family 7 member 11 (xCT) and glutathione peroxidase 4 (GPX4), thereby inducing ferroptosis and promoting BCa cell apoptosis (110). Dasatinib also inhibits the PI3K/AKT/mTOR pathway, downregulating Bcl-2 and p-AKT, while activating the caspase cascade and autophagy marker microtubule-associated protein 1A/1B-light chain 3 isoform II (LC3-II), inducing apoptosis and autophagy in BCa cells (111). Deoxyschizandrin targets arachidonate 5-lipoxygenase (ALOX5) expression, suppressing the phosphorylation of the PI3K/AKT pathway, and thereby inhibiting BCa cell proliferation, migration, and invasion, while inducing cell apoptosis (112). Additionally, phospholipase C epsilon (PLCϵ) was demonstrated to regulate glycolytic enzymes indirectly through the AKT/GSK3β/Cdc25a signaling cascade, promoting BCa metabolic reprogramming (122). In addition, ropivacaine, metformin, hesperetin, platycodin D, and cardamomin have also been shown to inhibit BCa progression through the PI3K/AKT pathway (113–117).

Recent studies have demonstrated that SRT1720, by facilitating SIRT1-mediated deacetylation of HIF-1α, significantly inhibits HIF-1α transcriptional activity and downregulates the expression of key glycolytic enzymes LDHA and PDK1, thereby inhibiting glycolysis and energy supply in BCa cells (118). Vitamin K2 activates the PI3K/AKT/HIF-1α pathway, promoting glycolysis while simultaneously inhibiting the TCA cycle, which induces metabolic stress and activates the AMPK/mTORC1 pathway, leading to autophagic cell death and suppression of BCa growth (119). With advancing research into the HIF-1α signaling pathway, drugs such as magnesium isoglycyrrhizinate and tanshinone IIA have been found to effectively inhibit BCa progression by modulating this pathway (120, 121).

In addition to the classic signaling pathways, recent studies have identified new metabolic regulators that serve as potential intervention targets. Protein phosphatase 3 catalytic subunit beta (PPP3CB), which plays a key role in regulating energy metabolism in normal tissues, is markedly reduced in BCa. It functions by directly binding to and promoting the degradation of pyruvate dehydrogenase kinase 1 (PDK1), thereby relieving its inhibition on the pyruvate dehydrogenase complex (PDC). This process facilitates the entry of pyruvate into the TCA cycle, ultimately reversing the Warburg effect and inhibiting tumor growth (123). Chondroitin polymerizing factor (CHPF) has recently emerged as a glycolysis-associated molecule in BCa. Its upregulation correlates with multiple glycolytic enzymes and transporters, while CHPF silencing is accompanied by changes consistent with reduced glycolysis and enhanced cuproptosis, suggesting a potential role in metabolic reprogramming and treatment response in BCa (124).

### 4.3 Translational barriers and clinical prospects of glycolysis-targeted therapy in BCa

Although glycolysis-targeted therapy has shown promising antitumor activity in BCa, most of the current evidence is still confined to preclinical models. As summarized in **Table 1**, both direct inhibition of key glycolytic enzymes and indirect modulation of upstream pathways can suppress glycolysis, inhibit tumor growth, and enhance chemosensitivity. However, the clinical translation of these strategies remains limited (125). This is consistent with the early clinical experience in BCa, where pathway-level metabolic interventions have shown only modest benefit in a subset of patients, rather than broad and durable efficacy.

Several factors may help explain this translational gap. First, toxicity and limited tumor specificity remain major challenges, as the metabolic pathways exploited by tumor cells are also essential for the function of normal tissues (126). Second, issues related to drug delivery and effective tumor-selective targeting have not yet been fully resolved (127). Third, the metabolic plasticity of BCa may further compromise the efficacy of single-pathway inhibition, because tumor cells can adapt to therapeutic pressure by reprogramming their energy metabolism and shifting toward alternative pathways (128). Moreover, the current literature suggests that the relationship between glycolysis inhibition and therapeutic efficacy is not always straightforward. Rather than acting through a single uniform mechanism, different agents may exert antitumor effects through partially overlapping metabolic, signaling, and stress-response pathways (129). This complexity may partly account for the variable biological outcomes reported across studies, even when similar glycolysis-targeting strategies are used.

Taken together, these observations suggest that the clinical value of glycolysis-targeted therapy in BCa will likely depend on biomarker-guided patient stratification and rational combination strategies, which may also help explain why metabolic monotherapy has not yet achieved major clinical success (128). In this context, glycolysis-related biomarkers, such as HK2, PFKFB3, serum LDH, and metabolic imaging parameters, may help identify metabolically vulnerable tumors and improve patient selection (11, 17, 130, 131). Future studies should therefore focus on improving tumor specificity, overcoming adaptive metabolic rewiring, and integrating glycolysis-targeted agents with chemotherapy, immunotherapy, or other metabolic interventions to enhance clinical applicability (132).

## 5 Conclusions and outlook

The study of metabolic reprogramming in BCa has offered new directions for clinical treatment, but it also presents several challenges. Strategies targeting key glycolytic enzymes and related pathways have shown promising antitumor potential, yet issues such as drug toxicity, metabolic plasticity, and treatment resistance still need to be addressed. For example, HK2 inhibitor 2-DG can effectively block glycolysis, but its systemic toxicity limits its clinical application (133). Similarly, PKM2 modulator mannose can induce tumor cell pyroptosis, but optimizing its delivery efficiency to reduce damage to normal cells still requires exploration (134). Therefore, future research needs to concentrate on enhancing the specificity of targeting while reducing toxicity to normal cells, such as developing precision delivery strategies based on nanoparticle drug delivery systems or exploring tissue-specific metabolic regulation methods. Combination therapies are also an inevitable choice to overcome metabolic plasticity and immune suppression. Efforts should focus on exploring the synergistic effects between glycolysis inhibitors and immune checkpoint blockade, chemotherapy, and targeted therapy. For instance, inhibiting lactate production could reverse TME immune suppression, thereby unleashing the cytotoxic potential of immune cells (135). Further combinatorial strategies could focus on the synergistic blockade of multiple metabolic pathways. For instance, a triple therapy simultaneously targeting the TCA cycle, phospholipid synthesis, and glycolysis has been proven to significantly suppress BCa growth by effectively inhibiting ATP production (136).

In terms of biomarker development, serum LDH levels have been reported to correlate with BCa staging, prognosis, and treatment response, making it a useful non-invasive clinical monitoring indicator (137). Additionally, although 18F-FDG PET/CT serves as a robust metabolic imaging modality that visually reflects tumor glycolytic flux (138), its clinical utility in NMIBC remains significantly constrained. The primary barrier is the physiological renal excretion of the 18F-FDG radiotracer, which leads to substantial radioactivity accumulation within the bladder lumen. This intense background signal severely obscures the detection of superficial or flat mucosal lesions, such as carcinoma in situ (139). Furthermore, the limited spatial resolution of standard PET/CT, combined with the inherently lower glycolytic activity of low-grade NMIBC, drastically reduces the target-to-background contrast (140). Consequently, while 18F-FDG PET/CT is highly efficacious for staging muscle-invasive and metastatic disease, the precise localized surveillance of NMIBC necessitates the development of alternative metabolic tracers or advanced functional imaging strategies (141).

To overcome current clinical bottlenecks in glycolysis-targeted therapy, future research must shift towards a higher-resolution understanding of BCa metabolism. The integration of spatial metabolomics with single-cell analysis is crucial for decoding single-cell metabolic heterogeneity within the TME (142). By utilizing multi-dimensional data, such as gene tags like Glycolysis.Sig constructed from pan-cancer single-cell cohorts (143), researchers can precisely map the dynamic aerobic glycolysis architectures of BCa. Furthermore, this high-resolution spatial mapping facilitates a deeper exploration of immunometabolism, which is essential to uncouple the intense glucose competition between tumor and immune cells, ultimately providing novel strategies to reverse TME immunosuppression and enhance the efficacy of targeted treatments (144).

Translating these complex mechanistic insights into clinical practice will increasingly rely on advanced data integration. Multi-omics modeling that integrates glycolytic profiles with clinical and pathological features will be indispensable. Supported by artificial intelligence (AI)-driven metabolic stratification, such multi-omics approaches can facilitate the development of predictive models capable of accurately forecasting prognosis and immunotherapy responses, thereby guiding precision patient management (145). A major challenge, however, is metabolic plasticity, as different molecular subtypes and disease stages may dynamically reprogram their glycolytic dependence under therapeutic pressure (146). Therefore, the robust validation of these dynamic glycolysis-related biomarkers in prospective large-sample cohorts remains a critical prerequisite.

Ultimately, the advancement of glycolysis-targeted therapy will depend not only on the discovery of novel metabolic targets but also on critical breakthroughs in drug delivery, toxicity management, and the clinical application of biomarkers. Currently, the core challenges lie in precisely delivering metabolic inhibitors to tumor tissues, minimizing systemic toxicity, and promoting biomarker-guided personalized treatment. Through the interdisciplinary integration of molecular biology, materials science, clinical medicine, and AI, targeted therapy for BCa is poised to overcome existing bottlenecks. This evolution will establish glycolysis-targeted intervention as a precise and efficient component of the comprehensive oncology treatment system, ultimately opening new avenues for improving patient prognosis and quality of life.
